# Global Calibration of Multiple Cameras Based on Sphere Targets

**DOI:** 10.3390/s16010077

**Published:** 2016-01-08

**Authors:** Junhua Sun, Huabin He, Debing Zeng

**Affiliations:** 1 Ministry of Education Key Laboratory of Precision Opto-mechatronics Technology, Beihang University, Beijing 100191, China; sjh@buaa.edu.cn (J.S.); binhuahe@163.com (H.H.); 2 Department of Applied Science and Technology and Center for Microplasma Science and Technology, Saint Peter’s University, Jersey City, NJ 07036, USA

**Keywords:** multiple vision sensors, global calibration, sphere targets, sphere center reconstruction

## Abstract

Global calibration methods for multi-camera system are critical to the accuracy of vision measurement. Proposed in this paper is such a method based on several groups of sphere targets and a precision auxiliary camera. Each camera to be calibrated observes a group of spheres (at least three), while the auxiliary camera observes all the spheres. The global calibration can be achieved after each camera reconstructs the sphere centers in its field of view. In the process of reconstructing a sphere center, a parameter equation is used to describe the sphere projection model. Theoretical analysis and computer simulation are carried out to analyze the factors that affect the calibration accuracy. Simulation results show that the parameter equation can largely improve the reconstruction accuracy. In the experiments, a two-camera system calibrated by our method is used to measure a distance about 578 mm, and the root mean squared error is within 0.14 mm. Furthermore, the experiments indicate that the method has simple operation and good flexibility, especially for the onsite multiple cameras without common field of view.

## 1. Introduction

Three-dimensional (3D) vision systems have the advantages of high precision and good flexibility, so they are widely applied in various fields. The multi-sensor vision system (MVS) is always used, because it has larger measurement range than a single sensor. The MVS needs onsite global calibration after being installed. As one of the most important technical indices of MVS, the measurement accuracy is directly influenced by the global calibration accuracy. In most practical applications of MVS, the structures of the measured objects and the environments are complex, and always lead to a complex distribution of the vision sensors. Vision sensors even have the feature of non-overlapping field of view (FOV), which requires that the global calibration methods have high precision and good flexibility.

Most classical global calibration methods [1,2,3,4,5] rely on matching features in the common FOV of all the sensors and are not applicable in the case of non-overlapping FOV. To overcome the limitations of non-overlapping FOV, Luo [6] used a two-theodolite system, and Kitahara *et al.* [7] used a 3D laser-surveying instrument to accomplish their global calibrations. They used precision auxiliary instruments to reconstruct the feature points in the world coordinate system (WCS) to acquire the transformation matrix between the camera coordinate system (CCS) and the WCS. However, when the on-site space is narrow, the auxiliary instruments have blind zones, and there is even might not be room for them. In the areas of video surveillance and motion tracking, Heng *et al.* [8], Pflugfelder *et al.* [9], Carrera *et al.* [10] and Esquivel *et al.* [11] used self-calibration methods. The cameras are globally calibrated through observing objects with specific structures in their FOV. In industrial measurement, there is little satisfactory scene information for the self-calibration process, and its accuracy usually cannot meet the requirements. Agrawal *et al.* [12], Takahashi *et al.* [13] and Kumar *et al.* [14] achieved the global calibration by making each camera view the targets in a mirror. However, no clear target is guaranteed to be observed by every camera in the complex MVS. The fixed constraints of multiple targets are used by Liu *et al.* [15] to calibrate multiple cameras. High accuracy can be achieved with this method, but the repeated pair-wise operations would reduce the calibration accuracy of MVS. Liu *et al.* [16] proposed a global calibration method based on skew laser lines. This method is flexible and can deal with the cameras with different viewing directions, but there are operational difficulties when we use it to calibrate multiple on-site cameras. Liu *et al.* [17] and De *et al.* [18] used a one-dimensional target to calibrate MVS, but it is hard to process a long one-dimensional target to calibrate vision sensors at a long distance.

Focusing on complexly distributed multi-camera systems with non-overlapping FOV, we present a global calibration method. It is based on several groups of spherical targets. A group of spheres is made up of at least three spheres without constraint. Each camera observes a group of spheres. At the same time, an auxiliary precision camera views all the spheres. The WCS coincides with the coordinate system of the auxiliary camera. Every camera to be calibrated and the auxiliary camera reconstruct the sphere centers of a group of spheres, so the transformation matrix from every CCS to the WCS can be calculated. The auxiliary camera is light and handy, and can be easily operated. Moreover, the sphere targets can be observed from different directions, so that any blind zones would be greatly reduced. Besides, this global calibration method can be realized through a one-time operation. It avoids the heavy workloads and accuracy losses caused by other repeated operations.

The paper is organized as follows: in Section 2, the calculation method of the sphere center reconstruction is first proved in detail. Then the calculation method of the transformation matrix is given, followed by the nonlinear optimization. Section 3 provides the accuracy analysis and experimental results. Both simulation and experimental data are provided to test and verify the presented technique. The conclusions are stated in Section 4.

## 2. Global Calibration Principle

**Figure 1:**
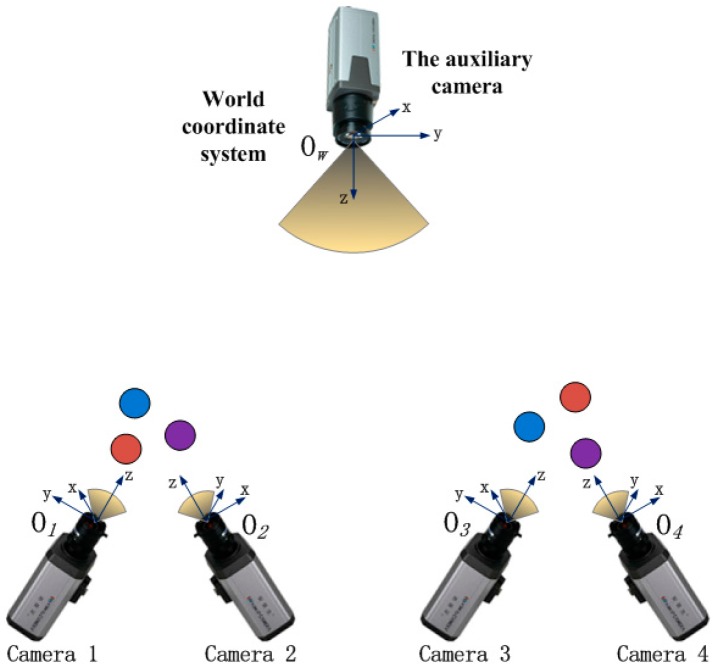
The principle of global calibration.

The principle of global calibration for multiple cameras is shown in Figure 1. Only two groups of cameras are drawn for the sake of discussion. There is no common FOV between the two groups of cameras to be calibrated, and each of them can observe at least three spherical targets. The auxiliary camera can view all of the sphere targets (at least six). If there are three or more groups of cameras to be calibrated, all the sphere targets should be visible to the auxiliary camera.

The global calibration process works as follows:

1. Install multiple cameras whose intrinsic parameters have been obtained. Then in the FOV of each camera, place at least three sphere targets. They should not be shaded by each other. Fix a precision auxiliary camera. It can view all the targets, and its coordinate system is regarded as the WCS.
2. Each camera takes a few images of the targets in its FOV. Then move the targets several times and repeat taking images.
3. Reconstruct the sphere centers of each group of spheres in the corresponding CCSs and the WCS. Then calculate the transformation matrix from every CCS to the WCS using nonlinear optimization. Thus the global calibration is completed.

### 2.1. Sphere Center Reconstruction

The general equation of ellipse includes five degrees of freedom. However, the ellipse in the sphere projection has two constraints, so it just has three degrees of freedom. Hence it can be represented by a parameter equation with only three parameters. The synthetic data show that the fitting accuracy of parameter equation is obviously higher than that of general equation. If appropriate parameters are chosen, the parameter equation is a linear expression of the three parameters. They can be acquired by linear least squares method. This calculation method is simple and has high accuracy.

In the following paragraphs, firstly, the equation F(*x*,*y*,*z*) = 0 of the conic surface with three parameters *λ*, *μ* and *σ* is established. Next, the equation f(*x*,*y*) = 0 of the ellipse curve is given according to the geometric relationship of the sphere projection. Then we calculate the three parameters *λ*, *μ* and *σ* through fitting a group of sampling points on the ellipse curve. Finally, the 3D coordinates of the sphere center in the CCS can be acquired from the three parameters.

#### 2.1.1. Sphere Projection Model

**Figure 2:**
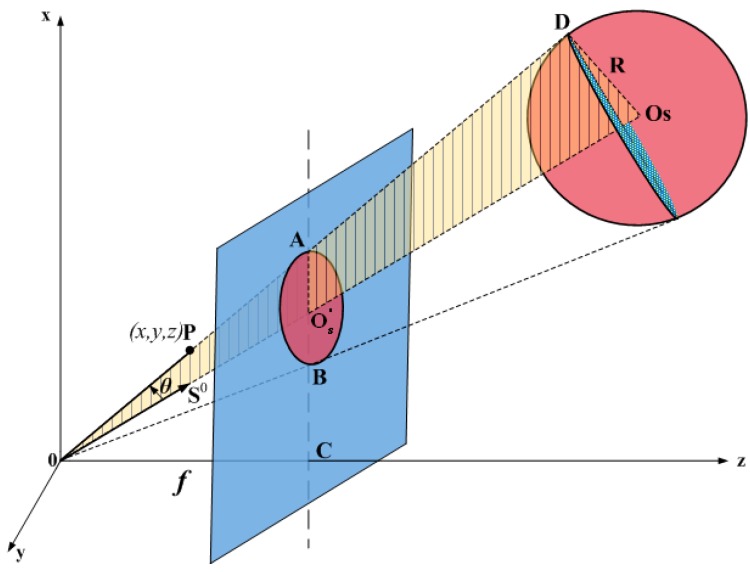
The sphere projection model.

In the following paragraphs, we propose a parameter equation F(*x*,*y*,*z*) = 0 to describe the sphere projection model. The geometric relationship of the sphere projection is shown in Figure 2. Point O is the center of the camera, and O-xyz is the CCS (in millimeters). Point C is the principal point of the camera and the origin of the image coordinate system (in millimeters). Its *x*-axis and *y*-axis are respectively in the same direction with the *x*-axis and *y*-axis of the CCS. Point O_S_ is the sphere center, and the point $O_{s}^{\prime }$ is an intersection point of the line OO_S_ and the image plane. The line OD and the sphere is tangent to the point D. Point A is an intersection point of the line OD and the image plane. The length of OC is the focal length *f*, and the sphere radius is known as R.

The sphere surface and the origin of the CCS, point O, can determine a conic surface. Its vertex is O. Each element of the conic surface is tangent to the sphere, and line OO_S_ is the symmetry axis of the conic surface. Let ∠DOO_S_ be *θ*, and the unit direction vector along OO_S_ be $S^{0}={\left[\cos\alpha ,\cos\beta ,\cos\gamma \right]}^{T}$ They satisfies the constraint $\cos^{2}\alpha +\cos^{2}\beta +\cos^{2}\gamma =1$. Let P(*x*,*y*,*z*) be any point of the conic surface, so that we have the equation of the conic surface as:

(1) $$\left(OP\cdot S^{0}\right)/\left|OP\right|=\cos\theta$$

Transforming Equation (1) to coordinate form and simplifying it, we have:

(2) $$\frac{\cos\alpha }{\cos\gamma }x+\frac{\cos\beta }{\cos\gamma }y+z-\frac{\cos\theta }{\cos\gamma }\sqrt{x^{2}+y^{2}+z^{2}}=0$$

Substituting three parameters: *λ =* cos*α*/cos*γ*, *μ =* cos*β*/cos*γ* and *σ =* cos*θ*/cos*γ* into Equation (2), we have the parameter equation of the conic surface in the CCS:

(3) $$F(x,y,z)=\lambda x+\mu y+z-\sigma \sqrt{x^{2}+y^{2}+z^{2}}=0$$

#### 2.1.2. Ellipse Curve Fitting

The intersection of the conic surface and the image plane *z* = *f* generates the ellipse curve, so that its equation in the CCS is:

(4) $$\{\begin{array}{c} \lambda x+\mu y+z-\sigma \sqrt{x^{2}+y^{2}+z^{2}}=0\\ z=f \end{array}$$

The origin of the image coordinate system is C and its *x*-axis and *y*-axis are respectively in the same direction with the *x*-axis and *y*-axis of the CCS. Eliminating the *z* in Equation (4), we have the ellipse equation in the image coordinate system:

(5) $$f(x,y)=\lambda x+\mu y-\sigma \sqrt{x^{2}+y^{2}+f^{2}}+f=0$$

If the normalized focal lengths of the camera are known as *f_x_* and *f_y_*, and its principal point is known as (*u*_0_,*v*_0_), we have:

(6) $$\frac{x}{f}=\frac{u-u_{0}}{f_{x}}\text{ and }\frac{y}{f}=\frac{v-v_{0}}{f_{y}}$$

where (*x*,*y*) (in millimeters) and (*u*,*v*) (in pixels) express the same point on the image plane. Substituting Equation (6) into Equation (5), we have the ellipse equation (in pixels):

(7) $$\lambda (\frac{u-u_{0}}{f_{x}})+\mu (\frac{v-v_{0}}{f_{y}})-\sigma \sqrt{{\left(\frac{u-u_{0}}{f_{x}}\right)}^{2}+{\left(\frac{v-v_{0}}{f_{y}}\right)}^{2}+1}+1=0$$

If {(*u_i_*,*v_i_*)_|_*i* = 1,2,3…,n} are known as the coordinates (in pixels) of a group of sampling points of the ellipse curve on an image, we can use linear least squares method to fit the ellipse curve, Equation (7). Then we acquire the optimal solutions of parameters *λ*, *μ* and *σ*.

#### 2.1.3. Sphere Center Coordinate Calculation

In the right triangle ODO_S_, we have $\left|OO_{s}\right|=R/\sin\theta$. Moreover, $S^{0}={\left[\cos\alpha ,\cos\beta ,\cos\gamma \right]}^{T}$ is defined as a unit direction vector along line OO_S_, so we have:

(8) $$Ο{Ο}_{s}=\left|Ο{Ο}_{s}\right|s^{0}=\frac{R}{\sin\theta }s^{0}={\left[\frac{R\cos\alpha }{\sin\theta },\frac{R\cos\beta }{\sin\theta },\frac{R\cos\gamma }{\sin\theta }\right]}^{T}$$

It is not hard to have the equation set:

(9) $$\{\begin{array}{c} \cos^{2}\alpha +\cos^{2}\beta +\cos^{2}\gamma =1\\ \lambda =\cos\alpha /\cos\gamma \\ \mu =\cos\beta /\cos\gamma \\ \sigma =\cos\theta /\cos\gamma \end{array}\Rightarrow \{\begin{array}{c} \sin\theta =\sqrt{1+\lambda^{2}+\mu^{2}-\sigma^{2}}/\sqrt{1+\lambda^{2}+\mu^{2}}\\ \cos\alpha =\lambda /\sqrt{1+\lambda^{2}+\mu^{2}}\\ \cos\beta =\mu /\sqrt{1+\lambda^{2}+\mu^{2}}\\ \cos\gamma =1/\sqrt{1+\lambda^{2}+\mu^{2}} \end{array}$$

Substituting equation set Equation (9) into Equation (8), we know **OO_S_** is ${\left[\frac{R\lambda }{\sqrt{1+\lambda^{2}+\mu^{2}-\sigma^{2}}},\frac{R\mu }{\sqrt{1+\lambda^{2}+\mu^{2}-\sigma^{2}}},\frac{R}{\sqrt{1+\lambda^{2}+\mu^{2}-\sigma^{2}}}\right]}^{T}$. Then we know the coordinates of the sphere center O_S_ in the CCS is $\left(\frac{R\lambda }{\sqrt{1+\lambda^{2}+\mu^{2}-\sigma^{2}}},\frac{R\mu }{\sqrt{1+\lambda^{2}+\mu^{2}-\sigma^{2}}},\frac{R}{\sqrt{1+\lambda^{2}+\mu^{2}-\sigma^{2}}}\right)$.

If the intrinsic parameters of the camera and the sphere radius are both known, the sphere center can be reconstructed from a single image. The sphere center coordinates reconstructed by multiple images can also be averaged to reduce the reconstruction error.

### 2.2. Transformation Matrix Calculation

**Figure 3:**
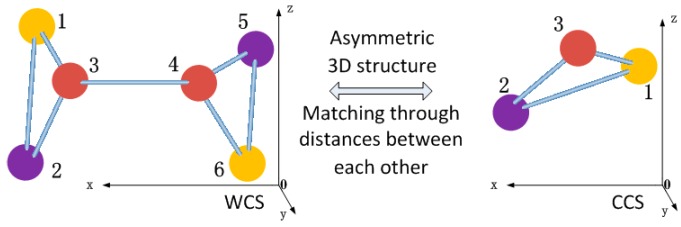
The sphere centers matching method.

As shown in Figure 3, all the spheres can be distributed to form an asymmetric 3D structure in the global calibration procedure. The distances between any sphere center and the others can form a signature vector. It can be used to distinguish this sphere center from the others. Through matching signature vectors, we match the sphere centers reconstructed in the CCS and the WCS.

In 3D space, the same sphere center can be described by two vectors, such as vector **P** in the CCS and vector **Q** in the WCS. In such a case, **P** and **Q** are called a pair of homonymous vectors. If three non-collinear sphere centers are reconstructed in both the CCS and the WCS, we get three pairs of homonymyous vectors. The transformation matrix between the CCS and the WCS can be calculated through them. Let three non-collinear sphere centers be described in the CCS as **P**_1_, **P**_2_ and **P**_3_, respectively, and **Q**_1_, **Q**_2_ and **Q**_3_ in the WCS. The transformation from the CCS to the WCS is defined as {**Q***i* = **R·P***_i_* + **T**_|_ *i* = 1,2,3…,n}, so the transformation matrix is $H=\left[\begin{array}{cc} R & T\\ 0 & 1 \end{array}\right]$.

The rotation matrix is calculated by:

(10) $$R=\left[Q_{1}Q_{2},Q_{2}Q_{3},Q_{1}Q_{2}\times Q_{2}Q_{3}\right]\cdot {\left[P_{1}P_{2},P_{2}P_{3},P_{1}P_{2}\times P_{2}P_{3}\right]}^{-1}$$

with $Q_{1}Q_{2}=Q_{2}-Q_{1}$, $Q_{2}Q_{3}=Q_{3}-Q_{2}$, $P_{1}P_{2}=P_{2}-P_{1}$ and $P_{2}P_{3}=P_{3}-P_{2}$. The translation vector

(11) $$T=(Q_{1}+Q_{2}+Q_{3}-RP_{1}-RP_{2}-RP_{3})/3$$

Consequently, the transformation matrix from the CCS to the WCS is acquired.

### 2.3. Nonlinear Optimization

In the practical calibration procedure, we acquire *n* pairs of homonymous vectors by fixing the cameras and placing the targets many times. Then the optimal solution of the transformation matrix **H** is calculated by using nonlinear optimization.

The objective function is:

(12) $$\min\;F={\sum_{i=1}^{n}\left\|{\overset{\sim }{Q}}_{i}-H{\overset{\sim }{P}}_{i}\right\|}^{2}$$

with ${\overset{\sim }{Q}}_{i}={\left[x_{q},y_{q},z_{q},1\right]}^{T}$ and ${\overset{\sim }{P}}_{i}={\left[x_{p},y_{p},z_{p},1\right]}^{T}$, where ${\overset{\sim }{Q}}_{i}$ and ${\overset{\sim }{P}}_{i}$ are respectively the homogeneous coordinates of $Q_{i}$ and $P_{i}$. Let the rotation matrix **R** be $\left[\begin{array}{ccc} r_{1} & r_{2} & r_{3}\\ r_{4} & r_{5} & r_{6}\\ r_{7} & r_{8} & r_{9} \end{array}\right]$, and it must satisfy orthogonal constraint. We have an equation set:

(13) $$\{\begin{array}{l} \begin{array}{c} h_{1}=r_{1}^{2}+r_{2}^{2}+r_{3}^{2}-1\\ h_{2}=r_{4}^{2}+r_{5}^{2}+r_{6}^{2}-1 \end{array}\\ h_{3}=r_{7}^{2}+r_{8}^{2}+r_{9}^{2}-1\\ h_{4}=r_{1}r_{4}+r_{2}r_{5}+r_{3}r_{6}\\ \begin{array}{c} h_{5}=r_{1}r_{7}+r_{2}r_{8}+r_{3}r_{9}\\ h_{6}=r_{4}r_{7}+r_{5}r_{8}+r_{6}r_{9} \end{array} \end{array}$$

Using the method of penalty function, from Equations (12) and (13) we get the unconstrained optimal objective function:

(14) $$\min F={\sum_{i=1}^{n}\left\|{\overset{\sim }{Q}}_{i}-H{\overset{\sim }{P}}_{i}\right\|}^{2}+M\sum_{j=1}^{6}h_{j}^{2}$$

where the penalty factor *M* determines the orthogonal error of the rotation matrix **R**. Here *M* takes a values of 10 considering the error distribution. Equation (14) is solved by the Levenberg-Marquardt method. The transformation matrix calculated from Equations (10) and (11) is used as the initial value of the iterations to solve Equation (14). Then we get the optimal value of the transformation matrix.

## 3. Analysis and Experiment

This section first discusses the factors which affect the calibration accuracy. The mathematical description and computer simulation are both given to analysis the calibration errors. Finally real data are given to evaluate the calibration accuracy.

### 3.1. Accuracy Analysis

The factors affecting the sphere center reconstruction are discussed here. According to Section 2.1.3, we have:

(15) $$oo_{s}=x\cdot i+y\cdot j+z\cdot k={\left[\frac{R\lambda }{\sqrt{1+\lambda^{2}+\mu^{2}-\sigma^{2}}},\frac{R\mu }{\sqrt{1+\lambda^{2}+\mu^{2}-\sigma^{2}}},\frac{R}{\sqrt{1+\lambda^{2}+\mu^{2}-\sigma^{2}}}\right]}^{T}$$

According to Equation (15), the partial derivative is:

(16) $$\frac{\partial oo_{s}}{\partial \lambda }=\frac{\partial x}{\partial \lambda }\cdot i+\frac{\partial y}{\partial \lambda }\cdot j+\frac{\partial z}{\partial \lambda }\cdot k$$

Let Δλ be the errors caused by noise, and $\Delta oo_{s}$ be the variation of the sphere center reconstruction, so we get:

(17) $$\Delta oo_{s}=\frac{\partial oo_{s}}{\partial \lambda }\cdot \Delta \lambda =\frac{\partial x}{\partial \lambda }\Delta \lambda \cdot i+\frac{\partial y}{\partial \lambda }\Delta \lambda \cdot j+\frac{\partial z}{\partial \lambda }\Delta \lambda \cdot k$$

Let $\left|\Delta oo_{s}\right|$ be the error of sphere center reconstruction, from Equation (17) we have:

(18) $$\left|\Delta oo_{s}\right|={\left[{\left(\frac{\partial x}{\partial \lambda }\Delta \lambda \right)}^{2}+{\left(\frac{\partial y}{\partial \lambda }\Delta \lambda \right)}^{2}+{\left(\frac{\partial z}{\partial \lambda }\Delta \lambda \right)}^{2}\right]}^{0.5}$$

From Equation (15), we the partial derivatives:

(19) $$\frac{\partial x}{\partial \lambda }=z-\frac{x^{2}z}{R^{2}},\;\frac{\partial y}{\partial \lambda }=-\frac{xyz}{R^{2}}\mathrm{and}\frac{\partial z}{\partial \lambda }=-\frac{xz^{2}}{R^{2}}$$

Substituting Equation (19) into Equation (18), we have:

(20) $$\left|\Delta oo_{s}\right|={\left[z^{2}+\frac{z^{2}x^{2}}{R^{4}}\left(L^{2}-2R^{2}\right)\right]}^{0.5}\Delta \lambda$$

where $L={\left(x^{2}+y^{2}+z^{2}\right)}^{0.5}=\left|oo_{s}\right|$, which means the distance between the sphere center and the camera center. When considering all the errors of Δλ, Δμ and Δσ, through the same proof procedure as above, we get:

(21) $$\left|\Delta oo_{s}\right|={\left[z^{2}+\frac{z^{2}x^{2}}{R^{4}}\left(L^{2}-2R^{2}\right)\right]}^{0.5}\cdot \Delta \lambda +{\left[z^{2}+\frac{z^{2}y^{2}}{R^{4}}\left(L^{2}-2R^{2}\right)\right]}^{0.5}\cdot \Delta \mu +{\left[\frac{z^{2}L^{2}}{R^{4}}\left(L^{2}-R^{2}\right)\right]}^{0.5}\cdot \Delta \sigma$$

From Equation (21), we conclude that the larger R, the smaller $\left|\Delta oo_{s}\right|$. The smaller *L*, the smaller $\left|\Delta oo_{s}\right|$. Therefore, the calibration accuracy can be improved by enlarging the radius of the sphere targets or putting the sphere targets closer to the camera.

### 3.2. Synthetic Data

In this section, we first analyzed some factors that affect the sphere center reconstruction accuracy. Then we analyzed the influence that the nonlinear optimization makes on the calibration accuracy.

#### 3.2.1. Factors That Affect the Sphere Center Reconstruction Accuracy

**Figure 4:**
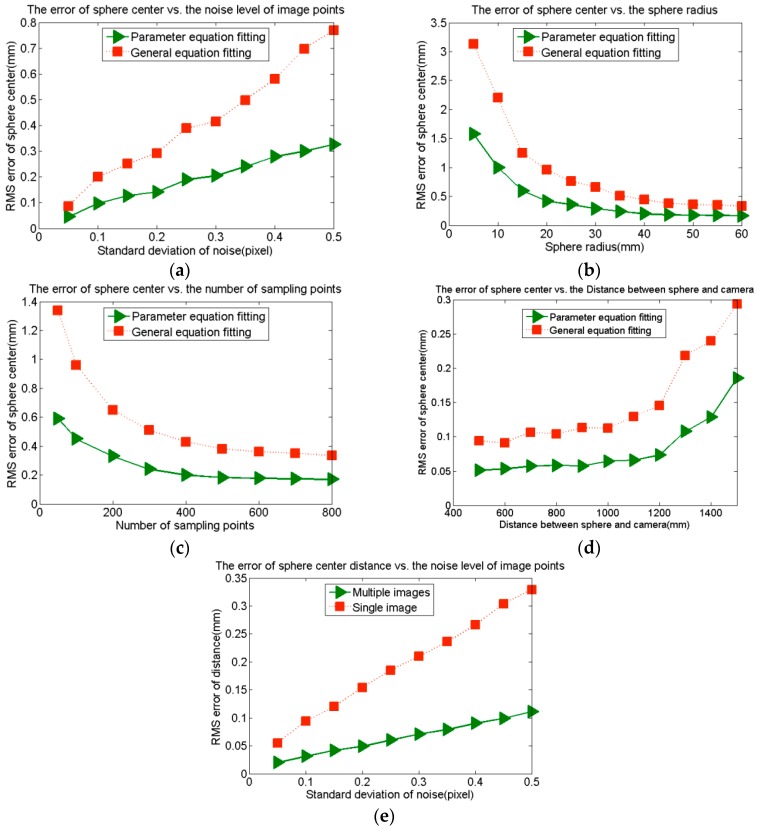
The effects of several major factors on the reconstruction error. (**a**) The noise level of image points; (**b**) The sphere radius; (**c**) The number of sampling points; (**d**) The distance between the sphere and camera; (**e**) Multiple images and a single image.

To verify the effectiveness of the sphere center reconstruction method, we use the software MATLAB to simulate it. The five factors include the ellipse fitting method, image noise level, sphere radius, sampling point number and distance between the sphere and camera. Their influences on the reconstruction error are studied. In the experiments, “general equation fitting” means using the general equation of the ellipse to fit it, and “parameter equation fitting” means using the parameter equation of the ellipse, Equation (7), to fit it. The experiment for each factor is repeated for 1000 times, and the reconstruction accuracy is evaluated by the root mean squared (RMS) error of the sphere center. The sphere radius is 40.000 mm in Figure 4a,c,d. The number of sampling points in Figure 4a,b,d is 600. Gaussian noise with 0 mean and 0.5 standard deviation is added to the image points in Figure 4b,c,d. The focal length is 24 mm and the image resolution is 1024 × 1024.

As shown in Figure 4a–d, improving the imaging quality, increasing the sphere radius, increasing the sampling point number and decreasing the distance between the sphere and camera can all improve the reconstruction accuracy. However, when the radius is more than 35 mm, the number of sampling points is more than 600 or the distance between the sphere and camera is less than 1000 mm, their influences are very small. Comparing the two fitting methods, we can conclude that the fitting accuracy of the parameter equation is higher than that of the general equation.

Finally, two spheres with constant distance are projected from four different directions to generate four images. The same Gaussian noise is added to each one. Each image is used to reconstruct the two sphere centers to calculate their distance, and the four distances are averaged as the simulation result of multiple images. The experiment is repeated 1000 times, and the reconstruction accuracy is evaluated by the RMS error of the sphere center distance. The sphere radius is 40.000 mm, and the number of sampling points is 600. The focal length is 24 mm, and the real distance between the two sphere centers is 400.000 mm. As shown in Figure 4e, the reconstruction accuracy of four images is higher than that of a single image.

#### 3.2.2. Effect of Nonlinear Optimization on Calibration Accuracy

In this subsection, we analyze the influence of the nonlinear optimization on the calibration accuracy through computer simulation. First, two cameras are globally calibrated by viewing three spherical targets at the same time, and the calibration results are calculated without optimization. Then, the two cameras are globally calibrated by viewing 15 sphere targets at the same time, and the calibration results are calculated through nonlinear optimization. The two kinds of results are compared to show the effect of optimization on the calibration accuracy. The intrinsic parameters of the two cameras are both set as:

(22) $$K=\left[\begin{array}{ccc} 2000 & 0 & 800\\ 0 & 2000 & 600\\ 0 & 0 & 1 \end{array}\right]$$

**Figure 5:**
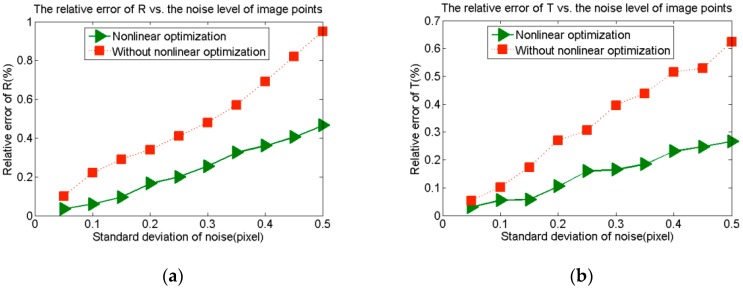
The effects of nonlinear optimization on the calibration accuracy. (**a**) The effects on the rotation vector; (**b**) The effects on the translation vector.

The position relationship of the two cameras are set as **R**_lr_ = [0.8, 0.02, 0.1]^T^ and **T**_lr_ = [–50, –400, –100]. The rotation vector is expressed as a Rodriguez vector here. The radius of the sphere targets is 40 mm, and the distance between the camera and the sphere center is about 1000 mm. The calibration accuracy is expressed by the relative error of **R**_lr_ and **T**_lr_. That is |Δ**R**_lr_|/|**R**_lr_| and |Δ**T**_lr_|/|**T**_lr_|. Gaussian noise with 0 mean and standard deviation (0.05–0.5) is added to the image points. The experiment is repeated for 1000 times, and the average of the relative error is regarded as the calibration error. As shown in Figure 5, the calibration results calculated through nonlinear optimization are more accurate than those without optimization.

### 3.3. Real Data

#### 3.3.1. Sphere Center Distance Measurement

In this experiment, the sphere radii and distances between the sphere centers have been accurately measured in advance as real values. A single camera is used to reconstruct two sphere centers to calculate their distance. In Section 2.1.3, we proved that a single camera can reconstruct the sphere center through a single image. When two spheres are both in the FOV of a camera, we use it to take an image of the two spheres, then the two sphere centers can be reconstructed through the image. If we get the 3D coordinates of the two sphere centers in the CCS, we can calculate their distance as the measurement result. Ten measurement results are used to calculate RMS error to evaluate the accuracy of sphere center reconstruction.

**Figure 6:**
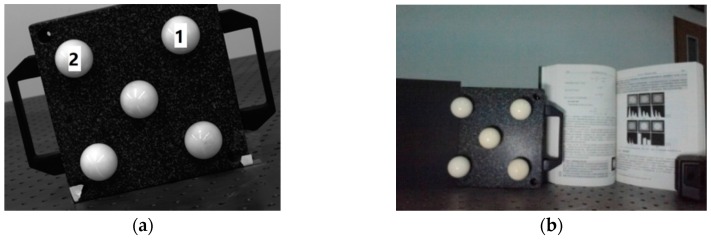
The experiment of sphere center distance measurement. (**a**) The positions and serial numbers of the targets; (**b**) A sample image for distance measurement.

The sphere targets and their serial numbers are shown in Figure 6a. The precise measurement shows that the diameters of sphere targets 1 and 2 are 40.325 mm and 40.298 mm, respectively, and the 3D coordinates of their centers are respectively (0.010, 0.019, 0.000) and (113.219, 0.022, 0.000). So that the standard value of the distance between the centers of spheres 1 and 2 is 113.229 mm.

The resolution ratio of the high-precision camera used in the experiment is 4256 × 2832, and its focal length is 24 mm, and its angle of view is 74° × 53°. The intrinsic parameters of this camera are:

(23) $$K=\left[\begin{array}{ccc} 2893.64571 & 0 & 2135.46395\\ 0 & 2900.50269 & 1401.48662\\ 0 & 0 & 1 \end{array}\right]$$

and “Plumb Bob” distortion model [19] is used to get its distortion coefficients:

(24) $$k_{c}=\left[0.09616,-0.07878,-0.00039,0.00006,-0.00896\right]$$

Forty images are captured and one of them is shown in Figure 6b. All the images are divided into 10 groups, and every group includes four images. Every image is used to calculate the distance between the centers of sphere 1 and 2. The four values calculated from every group of images are averaged, and the average value is regarded as a measurement result. All the ten measurement results are shown in Table 1.

**Table 1:** Measurement results of the sphere center distance.

Ten measurement results (mm)	113.282	113.246	113.413	113.329	113.312
	113.348	113.129	113.267	113.175	113.298
Average Value (mm)	113.280				
Real value (mm)	113.229				
RMS error (mm)	0.09				

#### 3.3.2. Global Calibration Results

**Figure 7:**
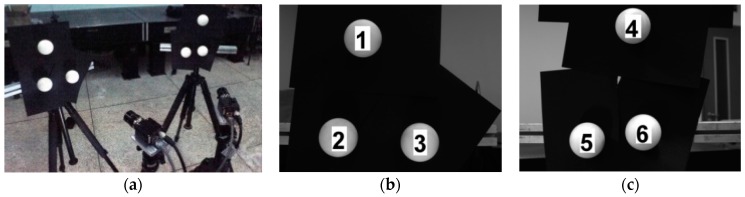
The global calibration experiment. (**a**) the physical system; (**b**) serial numbers of the targets viewed by the left camera; (**c**) serial numbers of the targets viewed by the right camera.

As shown in Figure 7a, two groups of sphere targets are used to calibrate two cameras without common FOV to verify the effectiveness of the global calibration method. Two groups of targets are respectively placed in the views of the two cameras. Each camera observes the targets in their own FOV, and an auxiliary precision camera observes all the sphere targets.

The targets in the experiment are white matte ceramic spheres. Their serial numbers are shown in Figure 7b,c. The diameters of the spheres are precisely measured in advance, and the results are shown in Table 2.

**Table 2:** Diameters of the sphere targets.

Serial Number	1	2	3	4	5	6
Diameter (mm)	50.700	50.720	50.715	50.708	50.710	50.723

The auxiliary camera used in the experiment is the camera used in Section 3.3.1 above, and its intrinsic parameters are shown as Equations (23) and (24). The cameras to be calibrated are common industrial cameras, whose resolution ratio is 1360 × 1024. Their intrinsic parameters are:

$$K_{\text{left}}=\left[\begin{array}{ccc} 1974.52417 & 0 & 728.88468\\ 0 & 1974.65442 & 549.29770\\ 0 & 0 & 1 \end{array}\right]$$

and:

$$K_{\text{right}}=\left[\begin{array}{ccc} 1977.18335 & 0 & 674.41864\\ 0 & 1976.94817 & 514.46720\\ 0 & 0 & 1 \end{array}\right]$$

respectively, and “Plumb Bob” distortion model [19] is used to get their distortion coefficients **k**_c_left_ = [–0.13109, 0.25232, –0.00007, 0.00018, 0.00000] and **k**_c_right_ = [–0.12748, 0.21361, –0.00000, 0.00002, 0.00000].

**Figure 8:**
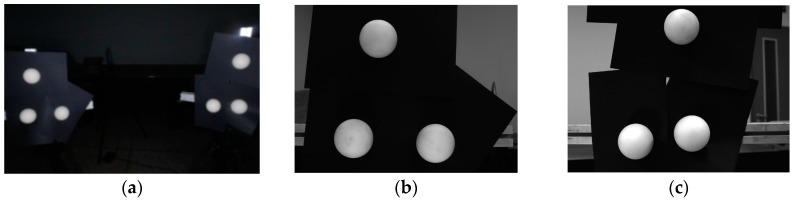
A group of sample images for the global calibration. (**a**) An image from the auxiliary camera; (**b**) An image from the left camera; (**c**) An image from the right camera.

All the cameras are fixed, and the two groups of sphere targets are placed for ten times in appropriate positions. Ten groups of images are captured and one of them is shown in Figure 8.

The transformation matrices from the left and right CCS to the WCS are respectively calculated using Equation (14). The results are:

(25) $$H_{\text{lw}}=\left[\begin{array}{cccc} 0.9319 & 0.3497 & 0.0968 & 25.057\\ -0.2239 & 0.7641 & -0.6050 & -180.185\\ -0.2855 & 0.5421 & 0.7903 & 389.945\\ 0 & 0 & 0 & 1 \end{array}\right]\text{ and }\;H_{\text{rw}}=\left[\begin{array}{cccc} 0.9581 & -0.0653 & -0.2788 & -11.453\\ 0.2442 & 0.6949 & 0.6763 & 204.524\\ 0.1496 & -0.7161 & 0.6818 & 413.723\\ 0 & 0 & 0 & 1 \end{array}\right]$$

Then the transformation matrix from the left CCS to the right CCS can be calculated from Equation (25). The global calibration result of the experiment is:

(26) $$H_{\text{lr}}=\left[\begin{array}{cccc} 0.7955 & 0.6027 & 0.0632 & -62.5153\\ -0.0120 & 0.1200 & -0.9927 & -252.7238\\ -0.6059 & 0.7889 & 0.1026 & -286.5962\\ 0 & 0 & 0 & 1 \end{array}\right]$$

#### 3.3.3. Global Calibration Accuracy Evaluation

**Figure 9:**
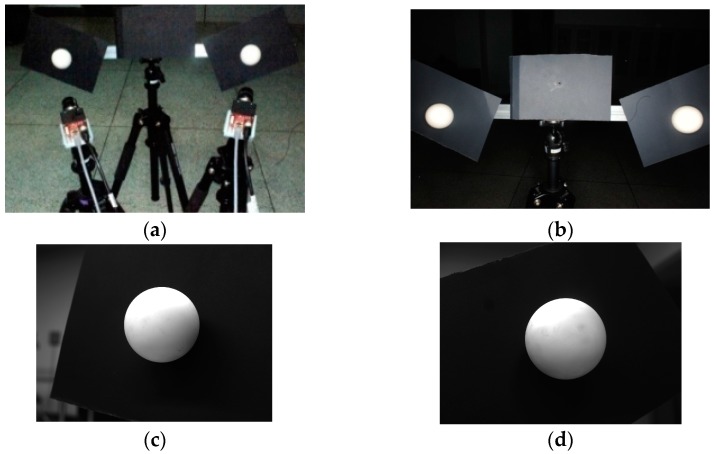
The experiment of global calibration accuracy evaluation. (**a**) The physical system; (**b**) An image from the auxiliary camera; (**c**) An image from the left camera; (**d**) An image from the right camera.

To verify the accuracy of the global calibration in Section 3.3.2 above, the two cameras make up a binocular vision system as shown in Figure 9a. Its global calibration result is shown in Equation (26). Sphere target 1 and sphere target 2 are fixed by a rigid rod, and are respectively laid in the views of the left and right cameras. Their diameters are shown in Table 2. The auxiliary camera measures the distance of the two sphere centers, and the average value of the results is regarded as the standard value of the distance. The two sphere centers are respectively reconstructed by the two cameras, so that their distance can be calculated based on the global calibration results. The distances measured by the binocular vision system are compared with the standard value to evaluate the accuracy of the global calibration.

One of the images captured by the auxiliary camera is shown in Figure 9b. The ten measurement results are shown in Table 3.

**Table 3:** Distances measured by the auxiliary camera.

Ten measurement results (mm)	578.004	578.154	578.091	578.043	578.291
	578.158	578.228	577.982	578.143	578.301
Average value $L_{0}$ (mm)	578.140				

The two sphere targets are placed in the right positions for ten times, and one group of the images captured by the binocular vision system is shown in Figure 9c,d. The ten distances measured by the binocular vision system and the EMS error are shown in Table 4.

**Table 4:** Distances measured by the binocular vision system.

Distance (mm)	578.068	578.191	578.227	578.254	578.264
	578.407	577.919	578.238	578.019	578.023
Real value $L_{0}$ (mm)	578.140				
Absolute error (mm)	−0.072	0.051	0.087	0.114	0.124
	0.267	−0.221	0.098	−0.121	−0.117
RMS error (mm)	0.14				

## 4. Conclusions

In this paper, we have developed a new global calibration method. In the calibration process, an isotropic sphere target can be simultaneously observed by different cameras from different directions, so the blind zones are reduced. There is no restriction on the position relationship between any two spheres, so the method is flexible in complex on-site environments. Moreover, a one-time operation can globally calibrate all the cameras without common FOV. This avoids the heavy workloads and accuracy loss caused by other repeated operations. A parameter equation is also used to fit the ellipse curve to improve the global calibration accuracy. Our experiments show that the proposed method has the advantages of simple operation, high accuracy and good flexibility. It can conveniently realize the global calibration of complexly distributed cameras without common FOV. In the practical application of this method, images with high-quality ellipse contours are necessary to the high accuracy of calibration.

## Abbreviations

The following abbreviations are used in this manuscript:

3D: three-dimensional
MVS: multi-sensor vision system
FOV: field of view
CCS: camera coordinate system
WCS: world coordinate system
RMS: root mean squared

## References

[1] Hu H., Liang J., Tang Z.Z., Shi B.Q., Guo X. (2012). Global calibration for multi-camera videogrammetric system with large-scale field-of-view. Opt. Precis. Eng..

[2] Wong K.Y., Zhang G., Chen Z. (2011). A stratified approach for camera calibration using spheres. Image Proc. IEEE Trans..

[3] Wang L., Wu F.C. (2007). Multi-camera calibration based on 1D calibration object. Acta Autom. Sin..

[4] Zhang Z.Y. (2000). A flexible new technique for camera calibration. IEEE Trans. Pattern Anal. Mach. Intell..

[5] Zhang H., Wong K.K., Zhang G.Q. (2007). Camera calibration from images of sphere. IEEE Trans. Pattern Anal. Mach. Intell..

[6] Luo M. (1996). Mutli-Sensors Vision Measurement System And Applications. Ph.D. Thesis.

[7] Kitahara I., Saito H., Akimichi S., Onno T., Ohta Y., Kanade T. Large-scale virtualized reality. Proceedings of the IEEE Computer Vision and Pattern Recognition(CVPR), Technical Sketches.

[8] Heng L., Furgale P., Pollefeys M. (2014). Leveraging Image-based Localization for Infrastructure-based Calibration of a Multi-camera Rig. J. Field Robot..

[9] Pflugfelder R., Bischof H. (2010). Localization and trajectory reconstruction in surveillance cameras with nonoverlapping views. IEEE Trans. Pattern Anal. Mach. Intell..

[10] Carrera G., Angeli A., Davison A.J. SLAM-based automatic extrinsic calibration of a multi-camera rig. Proceedings of the 2011 IEEE International Conference on Robotics and Automation (ICRA).

[11] Esquivel S., Woelk F., Koch R. (2007). Calibration of a multi-camera rig from non-overlapping views. Lect. Notes Comput. Sci..

[12] Agrawal A. Extrinsic camera calibration without a direct view using spherical mirror. Proceedings of the 2013 IEEE International Conference on Computer Vision (ICCV).

[13] Takahashi K., Nobuhara S., Matsuyama T. A new mirror-based extrinsic camera calibration using an orthogonality constraint. Proceedings of the 2012 IEEE Conference on Computer Vision and Pattern Recognition (CVPR).

[14] Kumar R.K., Ilie A., Frahm J.M., Pollefeys M. Simple calibration of non-overlapping cameras with a mirror. Proceedings of the IEEE Conference on Computer Vision and Pattern Recognition (CVPR).

[15] Liu Z., Zhang G., Wei Z., Sun J. (2011). A global calibration method for multiple vision sensors based on multiple targets. Meas. Sci. Technol..

[16] Liu Q., Sun J., Liu Z., Zhang G. (2012). Global calibration method of multi-sensor vision system using skew laser lines. Chin. J. Mech. Eng..

[17] Liu Z., Zhang G., Wei Z., Sun J. (2011). Novel calibration method for non-overlapping multiple vision sensors based on 1D target. Opt. Lasers Eng..

[18] De Franç J.A., Stemmer M.R., França M.B.M., Piai J.C. (2012). A new robust algorithmic for multi-camera calibration with a 1D object under general motions without prior knowledge of any camera intrinsic parameter. Pattern Recognit..

[19] Brown D.C. (1966). Decentering distortion of lenses. Photom. Eng..

